# The antibiotic resistance reservoir of the lung microbiome expands with age

**DOI:** 10.21203/rs.3.rs-3283415/v1

**Published:** 2023-09-18

**Authors:** Victoria T. Chu, Alexandra Tsitsiklis, Eran Mick, Lilliam Ambroggio, Katrina L. Kalantar, Abigail Glascock, Christina M. Osborne, Brandie D. Wagner, Michael A. Matthay, Joseph L. DeRisi, Carolyn S. Calfee, Peter M. Mourani, Charles R. Langelier

**Affiliations:** 1 Division of Infectious Diseases, University of California, San Francisco, CA, USA; 2 Chan Zuckerberg Biohub, San Francisco, CA, USA; 3 Chan Zuckerberg Initiative, San Francisco, CA, USA; 4 Division of Pulmonary and Critical Care Medicine, Cardiovascular Research Institute, University of California, San Francisco, CA, USA; 5 Department of Pediatrics, University of Colorado and Children’s Hospital Colorado, Aurora, CO, USA; 6 Department of Biostatistics and Informatics, Colorado School of Public Health, University of Colorado, Aurora, CO, USA; 7 Department of Biochemistry and Biophysics, University of California, San Francisco, CA, USA; 8 Arkansas Children’s Research Institute, Arkansas Children’s Hospital, Little Rock, AR, USA

**Keywords:** antimicrobial resistance, antibiotic resistance, resistome, lung microbiome, metatranscriptomics, aging

## Abstract

Antimicrobial resistant lower respiratory tract infections (LRTI) are an increasing public health threat, and an important cause of global mortality. The lung microbiome influences LRTI susceptibility and represents an important reservoir for exchange of antimicrobial resistance genes (ARGs). Studies of the gut microbiome have found an association between age and increasing antimicrobial resistance gene (ARG) burden, however corollary studies in the lung microbiome remain absent, despite the respiratory tract representing one of the most clinically significant sites for drug resistant infections. We performed a prospective, multicenter observational study of 261 children and 88 adults with acute respiratory failure, ranging in age from 31 days to ≥ 89 years, admitted to intensive care units in the United States. We performed RNA sequencing on tracheal aspirates collected within 72 hours of intubation, and evaluated age-related differences in detectable ARG expression in the lung microbiome as a primary outcome. Secondary outcomes included number and classes of ARGs detected, proportion of patients with an ARG class, and composition of the lung microbiome. Multivariable logistic regression models (adults vs children) or continuous age (years) were adjusted for sex, race/ethnicity, LRTI status, and days from intubation to specimen collection. Detection of ARGs was significantly higher in adults compared with children after adjusting for sex, race/ethnicity, LRTI diagnosis, and days from intubation to specimen collection (adjusted odds ratio (aOR): 2.16, 95% confidence interval (CI): 1.10–4.22). A greater proportion of adults compared with children had beta-lactam ARGs (31% (CI: 21–41%) vs 13% (CI: 10–18%)), aminoglycoside ARGs (20% (CI: 13–30%) vs 2% (CI: 0.6–4%)), and tetracycline ARGs (14% (CI: 7–23%) vs 3% (CI: 1–5%)). Adults ≥70 years old had the highest proportion of these three ARG classes. The total bacterial abundance of the lung microbiome increased with age, and microbiome alpha diversity varied with age. Taxonomic composition of the lung microbiome, measured by Bray Curtis dissimilarity index, differed between adults and children (p = 0.003). The association between age and increased ARG detection remained significant after additionally including lung microbiome total bacterial abundance and alpha diversity in the multivariable logistic regression model (aOR: 2.38, (CI: 1.25–4.54)). Furthermore, this association remained robust when modeling age as a continuous variable (aOR: 1.02, (CI: 1.01–1.03) per year of age). Taken together, our results demonstrate that age is an independent risk factor for ARG detection in the lower respiratory tract microbiome. These data shape our understanding of the lung resistome in critically ill patients across the lifespan, which may have implications for clinical management and global public health.

## Introduction

Antimicrobial resistance (AMR) is one of the top global health threats facing humanity^1^. Lower respiratory tract infections (LRTI) are a leading cause of death worldwide^1,2^, and account for a disproportionate burden of global AMR-related mortality, with an estimated 1.5 million deaths in 2019 attributable to resistant microbes^2^.

Despite the rise in AMR respiratory infections, the antimicrobial resistance genes (ARG) within the lung microbiome remain understudied and incompletely defined^3^. As with the gastrointestinal tract, the respiratory tract harbors diverse microbial communities acquired early during life^4–6^ that are continually influenced over the lifespan by exposures to organisms from the environment and other humans, as well as to antimicrobials. The gut, respiratory tract, and other human anatomical microbiomes serve as reservoirs for ARGs, or antimicrobial resistomes, and act as potential sites of ARG acquisition and transmission^7^.

An understanding of the epidemiological, biological, and clinical factors associated with AMR acquisition is crucial to halting the spread of resistant infections. Prior studies of the gut microbiome have demonstrated an association between age and the composition and burden of ARGs^8,9^, suggesting that cumulative exposures might shape the resistance landscape of endogenous microbial communities. Other factors influencing the gut resistome include travel^10^, hospital exposure^11^, and antibiotic use^12^. Despite these findings, corollary studies in the respiratory microbiome have not yet been performed, a key gap given the global magnitude of drug resistant LRTI. Furthermore, few studies have used metatranscriptomic RNA sequencing (RNA-seq) to both profile lower respiratory microbial ecology and detect ARG expression in the airway microbiome^3,13^.

Here, we sought to test the hypothesis that older age is associated with an increased prevalence of ARGs in the lung microbiome, using metatranscriptomics and multivariable logistic regression modeling. We find that age is indeed an independent risk factor for detecting ARGs in the lower airway microbiome, even after adjusting for multiple covariates including sex, race/ethnicity, LRTI diagnosis, community- versus hospital-acquired infection, days from intubation to specimen collection, and composition of the lung microbiome.

## Methods

### Study Design and Clinical Cohorts

We leveraged data from prospective pediatric^14–16^ and adult^17^ cohorts of patients with acute respiratory failure admitted to intensive care units (ICUs) in the United States (USA). Pediatric patients (n=261), aged 31 days to 18 years, were enrolled from eight tertiary care hospitals in the Collaborative Pediatric Critical Care Research Network (CPCCRN) between February 2015 and December 2017. Adults (n=88), aged >18 years, were enrolled from a single tertiary care center in California, USA between July 2013 to October 2017. From each enrolled patient, tracheal aspirates (TA) were collected within 72 hours of intubation, mixed with DNA/RNA shield, and stored at −80°C.

Electronic medical records were reviewed to obtain demographics and clinical data. LRTI status was retrospectively adjudicated by study physicians based on a previously described algorithm^16,17^, grouping patients as follows: 1) LRTI defined clinically, with or without a clinical microbiological diagnosis (LRTI); 2) No evidence of respiratory infection and a clear alternative etiology for the acute respiratory failure (No LRTI); or 3) patients who did not meet either above criteria (Indeterminate). LRTI was further separated into community-acquired LRTI (CA-LRTI; LRTI diagnosed within 48 hours of hospital admission), and hospital-acquired LRTI (HA-LRTI; LRTI diagnosed ≥48 hours after hospital admission).

### Metatranscriptomic RNA Sequencing, Taxonomic Alignment, and Detection of ARGs

RNA extracted from TA specimens underwent library preparation and paired-end Illumina sequencing, as previously described^16^. Quantification of microbial taxa from raw sequencing reads was carried out using the CZ-ID bioinformatics pipeline^18^, which performs reference-based alignment against microbial genomes from the National Center for Biotechnology Information (NCBI) nucleotide (NT) database, as previously described^18^. ARGs annotated in the Antibiotic Resistance Gene-ANNOTation (ARG-ANNOT) database^19^ were detected using the Short Read Sequence Typing (SRST2) algorithm^20^. Negative control water samples were processed in parallel, and a previously described negative binomial model was used to filter out microbial contaminants from the laboratory environment^16^. ARGs with <5% coverage or found in ≥10% of negative control water samples (TEM-1D, TetC, SulI, OXA-22, Aph3’Ia, CatA1) were excluded from the analysis.

### Statistical Analysis Framework

Age was defined in three ways: (1) a binary variable of children (31 days to 18 years) or adults (over 18 years); (2) nine subgroups of 0–2 years, 3–10 years, 11–18 years, 19–39 years, 40–49 years, 50–59 years, 60–69 years, 70–79 years, and ≥80 years; or (3) continuous age in years. We used Pearson’s Chi-square test for comparison of categorical variables. P-values <0.05 were considered statistically significant. All analyses were conducted in R (v4.2.1).

### Resistome Analyses

The number of ARGs detectably expressed in the lower respiratory tract microbiome of children and adults were compared at the individual gene and ARG class (e.g., beta-lactamase) levels. P-values were calculated using the Wilcoxon rank-sum test for nonparametric continuous variables and false discovery rate (FDR) correction was applied for multiple comparisons. We compared the proportion of detected ARG classes by binary age (pediatric versus adult) and by age subgroups. 95% confidence intervals [CI] for population proportions were obtained using the Clopper-Pearson exact binomial method.

ARG abundance was calculated based on the average sequencing read depth across each gene, normalized by gene length and total reads, reported as depth per million (dpm)^20,21^. Resistome alpha diversity was calculated using the Shannon Diversity Index (SDI) and ARG dpm. Beta diversity was calculated on patients with ARGs detected using the Bray-Curtis method with 1000 permutations using the PERMANOVA test and displayed via nonmetric multidimensional scaling (NMDS). Alpha and beta diversity calculations were performed using the R package *vegan*^22^.

A multivariable logistic regression model incorporating demographic and clinical characteristics (sex, race/ethnicity, LRTI status, days from intubation to specimen collection) was used to determine associations between binary age (adults vs children) and detection of ARGs Additional regression models were performed using: 1) age years as a continuous variable, and 2) the previously defined nine age subgroups. To assess for potential geographic differences in ARGs, an additional analysis was performed within the pediatric cohort only and included adjustment for U.S. census region and presence of complex chronic conditions; the latter was defined by a previously validated pediatric medical complexity algorithm^23^. A sensitivity analysis limited to pediatric and adult patients from the same U.S. census region was also performed. 95% confidence intervals (CI) for the multivariable logistic regression models were calculated using the Wald CI.

### Microbiome Analyses

We assessed the respiratory tract microbiome of children and adults to evaluate age-related differences in taxonomic composition and diversity, which we considered as possible confounders or mediators of the relationship between age and detectably expressed ARGs. We assessed microbiota at the genus level, calculated total bacterial abundance (measured in reads per million, rpM), and calculated bacterial alpha diversity across age subgroups. We further stratified by LRTI status (CA-LRTI, HA-LRTI, No LRTI). Lung microbiome beta diversity calculations were carried out using the Bray-Curtis dissimilarity index and PERMANOVA to assess statistical significance. Differential abundance analysis was performed using the R package *DESeq2*^24^ by assessing bacterial genera in the lung microbiome present in ≥20% of patients. We also described the prevalence of the most abundant species within each differentially expressed genus.

### Associations Between the Microbiome and Resistome Analyses

To test whether age-related differences in the lung microbiome might influence ARG results, we carried out additional analyses adjusting for bacterial abundance and alpha diversity. To test whether specific taxa might influence age-related changes in ARG detection, we performed a differential abundance analysis of bacterial genera detected in patients with or without detectable expression of ARGs, using *DESeq2*^24^. Subsequently, for each differentially abundant genus, we fit individual regression models for the outcome of having ARGs detected, accounting for bacterial abundance, alpha diversity, LRTI status, and presence of one of the differentially abundant genera. Lastly, additional sensitivity analyses were performed for these models using age as a continuous variable.

### Ethics

The pediatric cohort study was approved by a single Institutional Review Board (IRB) at the University of Utah (protocol #00088656). The adult cohort study was approved by the UCSF IRB (protocol #10–02701). Informed consent was obtained from parents or other legal guardians (pediatric patients) and from patients or their surrogates (adult patients), which included permission for collected respiratory specimens and data to be used in future studies. For the adult cohort, the IRB approved of an initial waiver consent for obtaining excess respiratory samples, and informed consent was subsequently obtained for continued study participation according to CHR protocol 10–02701 and as previously described^25^.

## Results

### Patient Cohorts

We studied 261 children (median age: 1 year, interquartile range (IQR): 0–15 years, range: 0–17 years), and 88 adults (median age: 63 years, IQR: 54–72 years, range: 21–94 years) (Supplemental Table 1). Of the 349 patients, 231 (66%) were adjudicated as LRTI-positive, 67 (19%) had no evidence of LRTI, and 51 (15%) of patients had indeterminate LRTI status. The proportion of patients in each LRTI adjudication group did not differ between the two cohorts. Adults had a higher proportion of HA-LRTI than children (25% vs 6%, respectively), emphasizing the need to include this as a covariate in our subsequent logistic regression model. In both cohorts, 90% of the patients received antibiotics prior to tracheal aspirate collection. All four U.S. census regions (Midwest, Northeast, South, West) in the U.S. were represented among the 261 pediatric patients; adult patients were from one enrollment site located in the regional West.

### Lower Respiratory Tract Resistome

**Figure 1.**
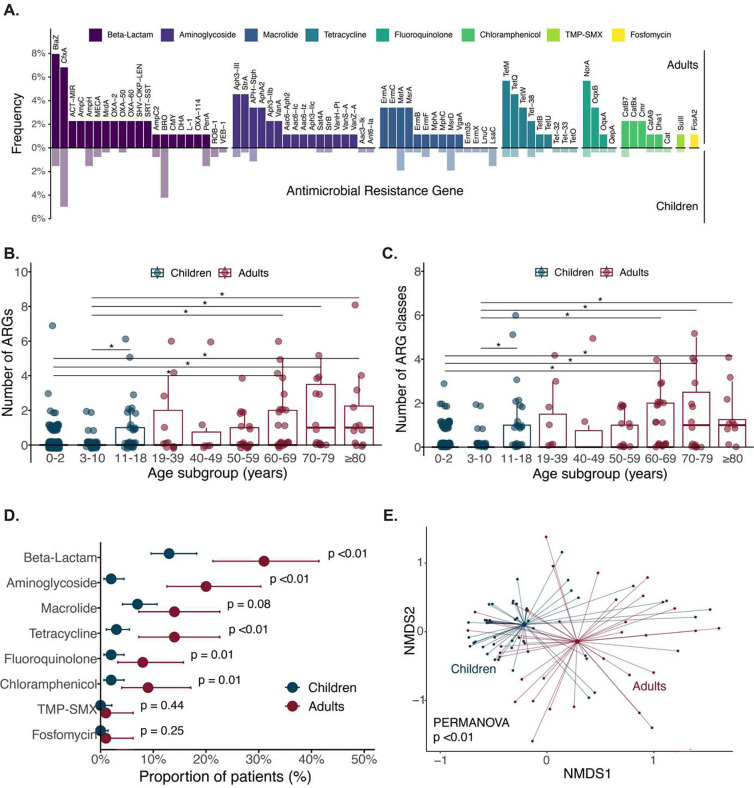
(**A**) Frequency of children (translucent) and adults (solid) with each antimicrobial resistance gene (ARG), stratified by ARG class. (**B**) Number of ARGs detected in children and adults by age subgroups. Two outliers were omitted for visualization purposes; one 11–18 year-old patient with 18 ARGs detected and another 70–79 year-old patient with 12 ARGs detected. (**C**) Number of ARG classes detected in children and adults by age subgroups. For Figures B and C, p-values were calculated using Wilcoxon-rank sum test and adjusted for multiple comparisons with False Discovery Rate (FDR) correction. The asterisks indicate statistically significant comparisons; all had a p-value <0.01. (**D**) Proportion of patients with ARGs by ARG class, stratified by pediatric and adult cohorts. The 95% confidence intervals were calculated by the Clopper-Pearson exact binomial method. P-values were obtained by Pearson’s Chi-square test and Fisher’s exact test for samples with <5 total ARGs. (**E**) Beta diversity of antimicrobial resistome children and adults. P-value calculated based on the Bray-Curtis dissimilarity index and the PERMANOVA test with 1000 permutations. Abbreviation: TMP-SMX, trimethoprim-sulfamethoxazole; NMDS, nonmetric multidimensional scaling.

ARGs were detectably expressed in the lower respiratory tract microbiome of 40 (45%) adults compared with 53 (20%) children (Pearson’s Chi-square p < 0.01). Across all patients, 74 distinct ARGs representing nine ARG classes were detected (Figure 1A). The number of detectably expressed ARGs (Figure 1B) and the number of ARG classes (Supplemental Figure 1) significantly differed between the youngest age subgroups (0–2 years and 3–10 years) and the oldest age subgroups (60–69 years, 70–79 years, and ≥80 years age groups), respectively. A significant increase was also noted between the 3–10 and the 11–18 years of age subgroups.

The most frequently detected ARG classes across all patients conferred resistance to beta-lactams (n=85 patients), macrolides (n=41), and aminoglycosides (n=37). A greater proportion of adults compared with children had beta-lactam ARGs (31% (CI: 21–41%) vs 13% (CI: 10–18%)), aminoglycoside ARGs (20% (CI: 13–30%) vs 2% (CI: 0.6–4%)), and tetracycline ARGs (14% (CI: 7–23%) vs 3% (CI: 1–5%)) (Figure 1C, Supplemental Table 2). When evaluated by age subgroup, the proportion of patients with beta-lactam, macrolide, or tetracycline ARGs was highest in patients ≥70 years of age (Supplemental Figure 2). Among young children, 13% (95% CI: 8–19%) of patients aged 0–2 years had a beta-lactam ARG compared with 2% (95% CI: 0.05–10%) of patients aged 3–10 years; this pattern was not seen for the other ARG classes. Among the beta-lactam ARGs, we detected six AmpC beta-lactamase genes, five extended-spectrum beta-lactamase genes, and 2 carbapenemase genes (Supplemental Figure 3).

ARG alpha diversity as measured by the Shannon Diversity Index increased primarily in patients ≥60 years of age (Figure 1D). The composition of the lung resistome significantly differed between children and adults, as measured by the Bray Curtis dissimilarity index (p = 0.003 by PERMANOVA) (Figure 1E).

**Figure 2.**
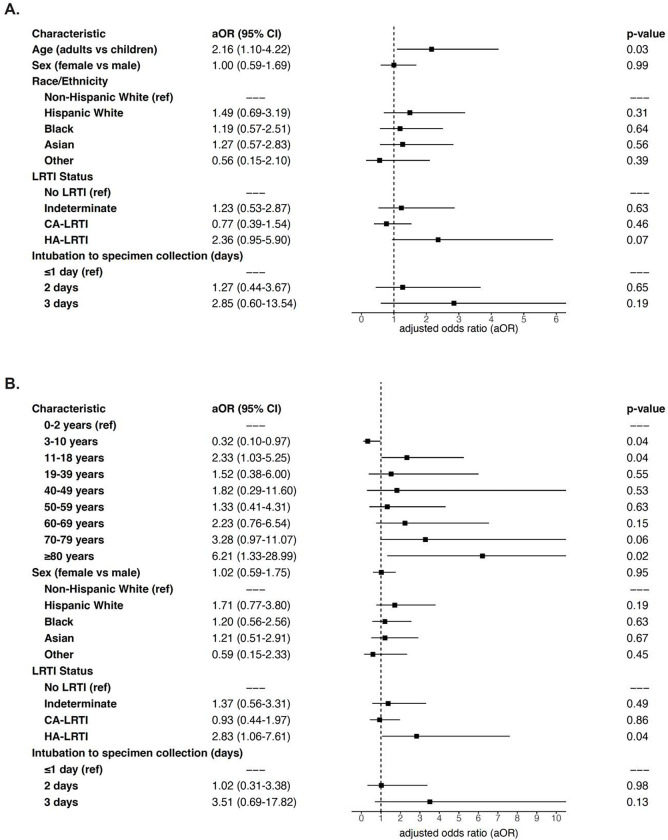
Multivariable logistic regression model evaluating the association of (**A**) binary age and (**B**) age subgroups with the presence of ARGs, accounting for sex, race/ethnicity, and lower respiratory tract infection (LRTI) status.

In a logistic regression model assessing the association of binary age group with detection of any ARGs, accounting for sex, race/ethnicity, LRTI status (CA-LRTI, HA-LRTI, No LRTI), and days from intubation to specimen collection, the risk of ARG detection was increased in adults compared with children (adjusted odds ratio [aOR]: 2.16, 95% CI: 1.10–4.22) (Figure 2A). Age remained significant in a sensitivity analysis of the same logistic regression model using age as a continuous variable (Supplemental Table 3). In a second sensitivity analysis using a regression model based on age subgroups (Figure 2B), children aged 3–10 years had a lower risk (aOR: 0.32, 95% CI: 0.10–0.97) and adults ≥80 years of age had a higher risk of detectably expressed ARGs (aOR: 6.21, 95% CI: 1.33–28.99) compared with children aged 0–2 years.

In an analysis restricted exclusively to children and accounting for enrollment U.S. census region and presence of a complex chronic condition, children 3–10 years of age continued to have the lowest risk of having detectably expressed ARGs, however enrollment site was a significant risk factor (Supplemental Table 4). Given this, we performed a sensitivity analysis limited to pediatric and adult patients from the same U.S. census region (West), and found that binary age group remained associated with increased risk of ARG detection (uOR: 2.33, 95% CI: 1.2–4.52).

### Lower Respiratory Tract Microbiome

**Figure 3.**
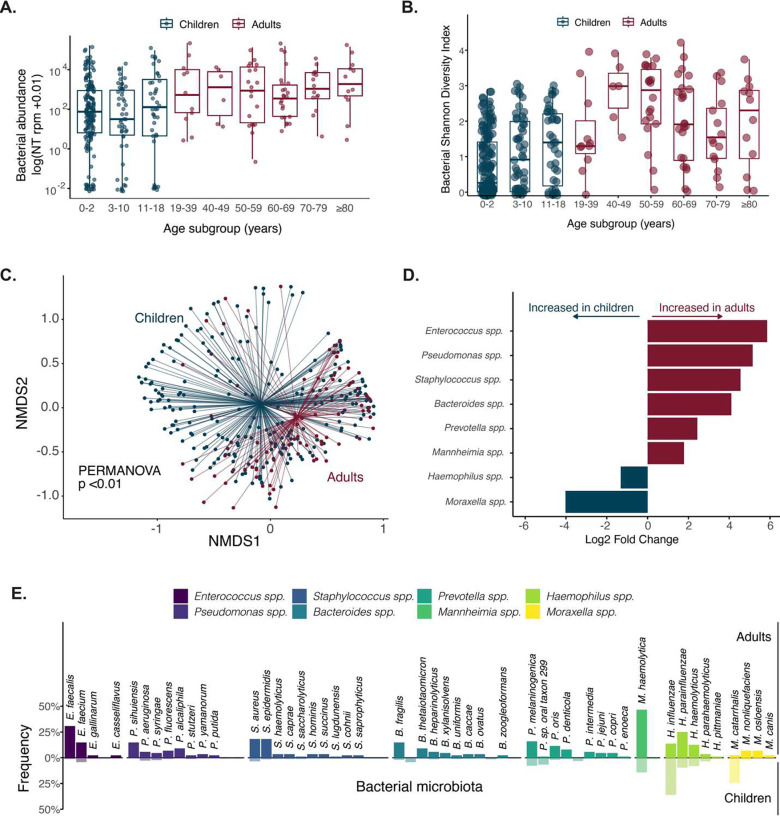
(**A**) Bacterial abundance in the lung microbiome measured in total bacterial alignments to the NCBI NT database per million reads sequenced (NT rpm) in children and adults by age subgroups. (**B**) Alpha diversity, calculated by the Shannon diversity index, of the bacterial lung microbiome of children and adults by age subgroups. (**C**) Beta diversity of the bacterial lung microbiome of children and adults. P-value calculated based on the Bray-Curtis dissimilarity index and the PERMANOVA test with 1000 permutations. (**D**) Statistically significant (p-value <0.05) differential abundant bacterial genera, by log_2_ fold change of bacterial counts, detected in children and adults. Bar colors indicate whether the species was more abundant in children (blue) or adults (red). (**E**) Frequency of the bacterial species detected in ≥5% of children (translucent) and adults (solid) among the differentially abundant bacterial genera. For patients with multiple species detected per genus, only the most abundant species was included in this analysis. Abbreviations: NT rpm, sequencing alignments to the NCBI NT database per per million reads sequenced; NMDS, nonmetric multidimensional scaling.

The total bacterial abundance of the lung microbiome increased with age (Figure 3A). Bacterial microbiome alpha diversity initially increased during childhood and adulthood, peaked in the 40–49 year-old age group, and then decreased in older adults (Figure 3B). The community composition of the bacterial respiratory microbiome differed between children and adults, based on Bray Curtis dissimilarity index (p < 0.01 by PERMANOVA) (Figure 3C). Differential abundance analysis revealed eight bacterial genera with statistically significant differences in abundance between children and adults (Enterococcus, Pseudomonas, Staphylococcus, Bacteroides, Prevotella, Mannheimia, Haemophilus and Moraxella). The most abundant bacterial species within each of these genera also differed between age groups (Figure 3E).

### Interactions Between the Lower Respiratory Tract Microbiome and Resistome

**Figure 4.**
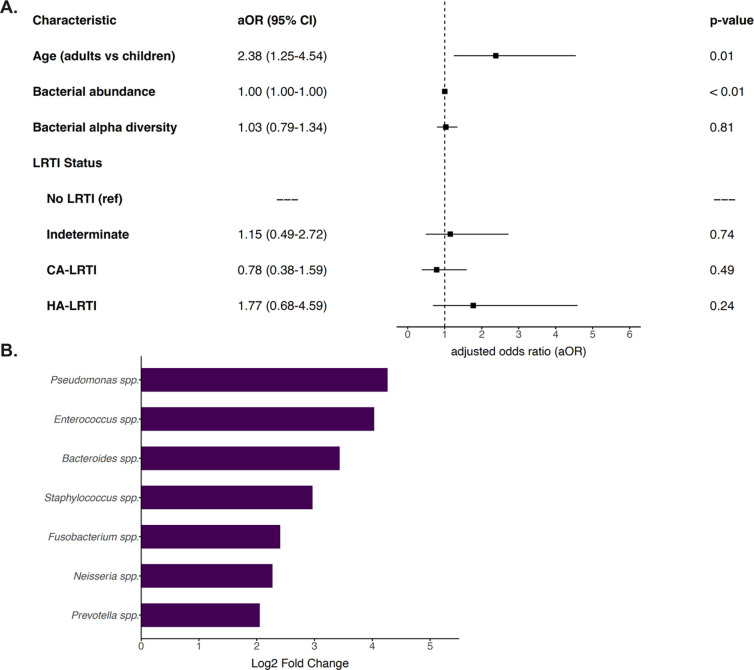
(**A**) Multivariable logistic regression model evaluating the association of binary age with the presence of ARGs, accounting for total bacterial abundance (NT rpm) per patient sample, bacterial alpha diversity. (**B**) Statistically significant (p <0.05) differentially abundant bacterial genera, by log2 fold change of bacterial counts, detected in patients with ARGs compared with patients without ARGs. All detected bacterial genera were more prevalent in patients with ARGs compared with patients without ARGs.

In a logistic regression model accounting for total bacterial abundance, bacterial alpha diversity, and LRTI status, binary age group remained associated with an increased risk of ARG detection (aOR: 2.38, 95% CI: 1.25–4.54) (Figure 4A). Differential abundance analysis revealed seven bacterial genera with statistically significant differences between patients with or without detectable ARGs (Figure 4B). In individual fitted logistic regression models where the outcome was presence of ARGs, and independent variables included age group, total bacterial abundance, bacterial alpha diversity, LRTI status, and one of the seven differentially abundant bacterial genera, adults remained associated with an increased risk of having ARGs detected compared with children (Figure 4A, Supplemental Table 5).

In these models, *Fusobacterium spp.* (aOR: 2.54, 95% CI: 1.32–4.90), *Bacteroides spp.* (aOR: 2.23, 95% CI: 1.15–4.33), *Enterococcus spp.* (aOR: 2.17, 95% CI: 1.04–4.51), *Staphylococcus spp.* (aOR: 2.09, 95% CI: 1.09–4.02), and *Prevotella spp.* (aOR: 1.98, 95% CI: 1.05–3.72) were statistically significant risk factors. Sensitivity analyses of all these models using age as a continuous variable demonstrated similar results (Supplemental Table 6).

## Discussion

Utilizing metatranscriptomics, we identify age as an independent risk factor for ARG detection in the lung microbiome among critically ill, recently intubated patients. We find that detection of ARGs in the lower respiratory tract increases across most of the age spectrum, with the oldest patients harboring the highest number of ARGs detectably expressed at the individual gene and class levels. These findings advance our understanding of the lung microbiome as a potential antimicrobial resistance reservoir and highlight its potential contribution to drug-resistant respiratory infections.

Across every ARG class examined, adults had a greater number of ARGs detected compared to children. The majority of detected ARGs in the pediatric cohort conferred resistance to beta-lactams and macrolides, while in adults, beta-lactam and aminoglycoside ARGs were most prevalent. Studies of nasal samples from healthy neonates^26^ and neonates with cystic fibrosis^27^ found a similar beta-lactam ARG dominance, while oral flora samples from children^28^ and sputum samples from adults^29^ found that macrolide resistance genes were most prevalent in the oropharynx. Intriguingly, we found that children 0–2 years of age had a higher proportion of beta-lactam ARGs detected than those 3–10 years of age. This may reflect maternally-derived microbial communities and associated ARGs acquired at birth, which go on to comprise the lung microbiome during the first years of life^30^.

Our findings differ from gut resistome studies which have found that tetracycline ARGs are most prevalent, followed by macrolides and beta-lactam ARGs^31^. These differences may reflect differences in the routes of ARG and antibiotic exposure (e.g., inhaled, ingested, intravenous) and highlight potential AMR transmission differences from the lungs compared with the gut. Indeed, recent work demonstrates the presence of ARGs and microbiota in urban air samples^32,33^, suggesting that environmental exposures may be a relevant route of lung resistome exchange particularly for patients with prolonged or repeated exposure to healthcare facilities where resistant microbes are more prevalent.

In the U.S., the most prescribed outpatient antibiotics are beta-lactams, fluoroquinolones, and macrolides^34^, while beta-lactams, macrolides, and glycopeptides are the most frequently prescribed inpatient antibiotics^35^. While beta-lactam ARGs were the most prevalent ARG class detected in both children and adults, adults had a greater proportion of aminoglycoside and tetracycline ARGs. Given that these antimicrobial classes have been widely used in the agriculture and livestock industries^36^, exposures to environmental bacteria harboring these ARGs over the lifespan could be one possible explanation. Other possible explanations include community exposures to other individuals, co-selection of ARGs on mobile genetic elements carrying multiple ARGs, or cross-resistance due to multi-drug efflux pumps^37^.

We also observed differences in the lower respiratory tract microbiome with age, including bacterial abundance, diversity, and taxonomic composition. Our findings are in line with a prior study demonstrating that bacterial abundance in the lung microbiome of CF patients increases with age^38^. We also found that bacterial alpha diversity increased in childhood, peaked in middle age, and decreased with older age. The role of endogenous respiratory microbiota in both the pathophysiology and diagnosis of critical illness syndromes in increasingly recognized, and our results suggest that adjusting for age should be considered in clinical and translational studies of the lung microbiome.

Even when accounting for demographic, clinical, and microbiome differences, age remained an independent risk factor for ARG detection. Our findings raise the possibility that selective environmental pressures driving AMR acquisition from the environment may be continuous over the lifespan, shaping the airway microbiome and associated resistome. While the detection of ARG expression in the airway microbiome does not equate to clinically relevant resistance, it suggests the potential for development of phenotypic resistance^3^ with possible implications for patient care. Commensal bacteria within the lung microbiome can exchange ARGs via horizontal gene transfer to pathogens or pathobionts, leading to the emergence of drug resistant LRTI^39–41^. Further research is needed to better characterize and understand the prevalence, acquisition, and transmission dynamics of ARGs within the lung microbiome.

This study has limitations. First, there was not an even distribution of patients across all ages, with a greater number of young children and older adults, reflecting the distribution of the critically ill, mechanically ventilated patient populations. To account for this, we performed sensitivity analyses using age as a continuous variable or using age subgroups. Second, there were differences in geographic location and timing of tracheal aspirate collection between age groups, which we accounted for by incorporating the variables into the multivariable logistic regression models. Third, the study included only patients from the U.S. and may not be representative of the global population. Fourth, detection of ARGs is biased towards the most abundant taxa in the lung microbiome and we are likely missing detection of ARGs from less abundant taxa. Finally, enrollment occurred prior to the COVID-19 pandemic. Thus, ARG abundance and class profiles may be different than in the current population given the increase in antibiotic use and AMR infections since 2020^42,43^.

In summary, we demonstrate that age is independently associated with the detection of ARGs in the lung microbiome in a population of critically ill patients soon after intubation. Our results suggest that healthcare, community, and environmental exposures throughout life may contribute to the reservoir of ARGs in the respiratory tract. Taken together, these findings advance our understanding of AMR in the context of the human microbiome and have implications for the management of infectious diseases, antimicrobial stewardship programs, and public health policies.

## Data Availability

FASTQ files containing non-host reads identified by the CZ-ID pipeline, following subtraction of reads aligning to the human genome, are available in the NCBI Sequence Read Archive (SRA) database under BioProject accessions PRJNA875913 and PRJNA450137. All data, code, and results are available at: https://github.com/victoriatchu/agingAMR.
